# Music as a manifestation of life: exploring enactivism and the ‘eastern perspective’ for music education

**DOI:** 10.3389/fpsyg.2015.00345

**Published:** 2015-03-27

**Authors:** Dylan van der Schyff

**Affiliations:** Faculty of Education, Simon Fraser University, Burnaby, BCCanada

**Keywords:** enaction, embodied music cognition, holistic music education, Buddhist psychology, life philosophy, music and language, music perception, music and culture

## Abstract

The enactive approach to cognition is developed in the context of music and music education. I discuss how this embodied point of view affords a relational and bio-cultural perspective on music that decentres the Western focus on language, symbol and representation as the fundamental arbiters of meaning. I then explore how this ‘life-based’ approach to cognition and meaning-making offers a welcome alternative to standard Western academic approaches to music education. More specifically, I consider how the enactive perspective may aid in developing deeper ecological understandings of the transformative, extended and interpenetrative nature of the embodied musical mind; and thus help (re)connect students and teachers to the lived experience of their own learning and teaching. Following this, I examine related concepts associated with Buddhist psychology in order to develop possibilities for a contemplative music pedagogy. To conclude, I consider how an enactive-contemplative perspective may help students and teachers awaken to the possibilities of music education as ‘ontological education.’ That is, through a deeper understanding of ‘music as a manifestation of life’ rediscover their primordial nature as autopoietic and world-making creatures and thus engage more deeply with musicality as a means of forming richer and more compassionate relationships with their peers, their communities and the ‘natural’ and cultural worlds they inhabit.

## Introduction

In recent years there has been a growing interest among music psychologists and philosophers of music and music education to explore the embodied and world-making aspects of human musicality (DeNora, 2000; Bowman, 2004; Reybrouck, 2005; Krueger, 2009, 2011a,b; Elliott and Silverman, 2014). This has involved a critical decentering of Cartesian models of cognition, Enlightenment esthetics, and related Western academic assumptions of what music and music education entail (Small, 1998; Johnson, 2007). Indeed, it is increasingly recognized that affective-conative and synergistic activities such as musicking afford pre-rational openings to empathic, embodied and ethical ways of knowing and being when our ‘inner’ realities shape, and are shaped by, those of others–thus highlighting the deep continuity between movement, feeling and the ‘extended’ or interpenetrative nature of cognition and ‘mind’ (Mathews, 2008; see also Clark and Chalmers, 1998; Nakagawa, 2000; Menary, 2010; Colombetti, 2014).

This renewed interest in the deep relevance of musicality for human development and well-being is revealing music as a ‘focal practice’ that supports a total ‘ontological’ education (Thomson, 2001; Dreyfus, 2004). This means that a music educator dedicated to developing a wide range of interdisciplinary knowledge in a critically reflective, praxis-based context may accomplish much more than getting students to develop technical fluency or to listen and perform according to pre-given standards and conventions (Elliott and Silverman, 2014). She may help students to see music not merely as a thing to be reproduced or consumed, as a product or a ‘pleasure technology’ (Pinker, 2009), but rather as an opening to the world and with it a range of scientific, philosophical, ecological, ethical, socio-cultural and ‘self’ knowledge not traditionally associated with music education in the West. Along these lines, an ontological music education may also play an important role in developing much needed forms of global ecological and ‘holistic’ thinking that look beyond the reductive, reifying, instrumental, and commodifying tendencies often associated with the Western perspective (Bai, 2001; Kincheloe, 2003, 2008; Giroux, 2011).

With this in mind I discuss here the relevance of what Nakagawa (2000) refers to as the ‘Eastern perspective’ ^1^ for (music) education. Despite the geographical denotation, this view is not limited to Asian philosophy and psychology. Rather it encompasses a range of thinkers, Eastern and Western, who in various ways understand mind and consciousness as fundamentally embodied and ecological phenomena; and who discuss ontological issues in the context of contingency, transformation and the interpenetration of dynamic systems. Over the past decades this perspective has become associated with the interdisciplinary research program known as enactivism, ^2^ which draws on Buddhist psychology as well as thinkers associated with Western cognitive science, phenomenology, and existential philosophy (Varela et al., 1993; Thompson, 2007). As I go, I develop this general perspective in the context of a life-based philosophy of music education in order to decentre the Western focus on objectivist or third person didactic approaches. To conclude, I consider possibilities of a contemplative-enactive music pedagogy for (re)connecting students and educators with their lived, embodied realities (Bai, 2003).

### The Biological Origins of Mind and Meaning

Generally speaking, the ‘Eastern perspective’ may be understood in the context of a holistic ‘life philosophy’ (Miller, 1997; Nakagawa, 2000). As its name suggests, life philosophy embraces an ‘animate’ ontology (Bai, 2013) and thus a pedagogical practice based in such an approach “conceives of education as an integral part of the greater Life processes; that is, education is a manifestation of Life and at the same time a vehicle in the service of reconnecting human life with the fundamental life” (Nakagawa, 2000, p. 79). This means that an ‘Eastern’ approach to music education requires a radical opening up to the fundamental organic processes that afford communication and meaning-making beginning at the most primordial levels of embodied being-in-the-world.

In order to better understand what this entails we may begin by considering the work of a range of ecological and life-minded thinkers who understand cognition and ‘mind’ as a relational or ‘enactive’ process (Bateson, 1972; Varela et al., 1993; Thompson, 2007; Stewart et al., 2010). This perspective understands creative, living cognition not as a distinct disembodied category or in terms of dualistic-mechanistic Cartesian metaphors (e.g., the mind as computer). Instead, it sees mind as a relational process that is ontologically continuous with basic processes of life itself ^3^. Here communication and meaning-making are explored in terms of the deep relationship between action and perception–where a meaningful world is ‘brought forth’ or ‘enacted’ from a background of understanding that develops through an ongoing history of structural coupling between organism and environment (Varela et al., 1993; O’Regan and Nöe, 2001; Nöe, 2006). Biologist Richard Lewontin explains,

Just as there is no organism without an environment, so there is no environment without an organism. The organism and environment are not actually separately determined. The environment is not a structure imposed on living beings from outside but is in fact a creation of those beings. The environment is not an autonomous process but a reflection of the biology of the species. (Lewontin, 1983, p. 99)

And therefore,

[...] there is always a ‘next step’ for the system in its perceptually guided action […] the actions of the system are always directed toward situations that have yet to become actual. Thus cognition as embodied action both poses the problems and specifies those paths that must be tread or laid down for their solution. (Lewontin, 1983; also quoted in Varela et al., 1993, p. 205)

Put simply, this conception of cognition allows us to consider organism and environment not as a pre-given duality, but rather as dependently co-arising through the activity of the organism as it brings forth a world. By this light, living cognition is necessarily contextually adaptive and creative–like a “path that exists only as it is laid down in walking” (Varela et al., 1993, p. 205). Thus while the enactive approach asserts the inseparability of the organism and environment, it also highlights the organism’s autonomy–or its ability to enact a world in ways that are not completely driven from the side of the environment. This means that the organism-environment relationship is necessarily asymmetrical; that *living* cognition is based, first and foremost, in the affectively motivated (valenced) sensory-motor activity of organic systems that exhibit ‘operational closure’ (Varela, 1979; Di Paolo, 2005, 2009; Thompson, 2007; Colombetti, 2014).

In order to better understand what this entails, one might consider how the input–output functions of a computer depend on externally imposed designs (hardware and software), information processing rules (system language), and interpretations of outputs; it is not self-making and thus cannot function meaningfully in an autonomous fashion; its operations are dependent on, and must remain open to, the external (human) forces that impose meaning, functionality and form. In living systems, however, the meaning of this or that interaction is “not prescribed from outside but is the result of the organization and history of the system itself” (Varela et al., 1993, p. 157). Here it is important to recognize that while the meaningful world enacted by the organism-environment couplings of living systems are ‘operationally closed’ (intrinsically meaningful), the relationship between organism and environment must remain dynamically open so that the ‘information’ developed by the system maintains its contextual relevance (Bateson, 1972). As Thompson puts it,

[…] information, dynamically conceived, is the making of a difference that makes a difference for some-*body* somewhere. Information here is understood in the sense of *informare*, to form within. An autonomous system becomes informed by virtue of the meaning formation in which it participates, and this meaning formation depends on the way its endogenous dynamics specifies things that make a difference to it. (Thompson, 2007, p. 57)

By this light, information does not first consist of abstract symbols, nor is it simply ‘out there’ to be anonymously processed. Rather it is ontogenic*–*it grows from the relevant relationships and valences (‘primordial dynamism’; Thompson, 2007) that emerge as a dynamic organism-environment system constitutes a life-world (Oyama, 2000). Thus cognition and meaning-making are not first understood as rule-based ‘problem solving’ on the basis of optimizing correspondence with a pre-given world, but rather as an ongoing creative process through which a viable or ‘sufficing’ world is brought forth (Varela et al., 1993; Di Paolo et al., 2010; see also Merleau-Ponty, 2002; Heidegger, 2008).

Put simply, this mode of meaning-making goes deeper than fact-based knowledge, technical knowledge or knowing ‘this or that.’ Rather, it is based in the adaptive and embodied learning processes that enable, “knowing how to negotiate our way through a world that is not fixed and pre-given but that is continually shaped by the types of actions in which we engage” (Varela et al., 1993, p. 144). Thus, given the variety of transforming environments in which human beings and other organisms live and interact, the enactive approach embraces the consistencies of experience, but also, crucially, the differences. As Bateson (1972, 1979/1980) points out, it is this ability to perceive and communicate the “news of difference” that binds the living world together ^4^. And indeed, it is just this conative, affective, and communal reaching out to (and mutual transformation through) difference that characterizes the asymmetry of the dynamic organism-environment relationship I began to discuss above–whereby a basic metabolic perspective of value, a point of view, or indeed, a ‘self’ may arise, develop and flourish (Jonas, 1966; Di Paolo, 2005; Thompson, 2007; Barbaras, 2010) ^5^.

As Maturana and Varela (1980, 1992) demonstrate, such *autopoietic* (self-making) histories of dynamic organism-environment couplings may be observed in even the simplest single celled organisms. While such creatures clearly do not possess the neural complexity to support abstract representations, they nevertheless move purposefully, communicate, develop viable relationships, and thus maintain a life-world in the transforming environments they inhabit. Although it would certainly be premature to understand such creatures as conscious, these basic forms of life nevertheless exhibit the origins of cognition and mind as valenced, affective, perceptually guided action (Thompson, 2007; Colombetti, 2014).

Here we may also consider how such simple autopoietic organisms may reach out to each other and thus function collectively as interpenetrative dynamic environments–resulting in more complex systems such as multi-celled organisms; nervous, respiratory, and immune systems; brains; social organizations; and the emergence of ‘reason,’ language, culture, and consciousness (Varela et al., 1993; Oyama, 2000; see also Johnson, 2007; Froese and Di Paolo, 2011). It follows, then, that for highly complex organisms such as human beings autopoiesis entails a lived developmental history including social or bio-cultural embodiment within a domain of “consensual action and cultural history” (Varela et al., 1993, p. 149; see also Hutchins, 2010; Cuffari et al., 2014) ^6^. With this in mind, we may begin to consider musicality as a manifestation of such inter(en)active life processes. That is, as a fundamental participatory (De Jaegher and Di Paolo, 2007) sense-making capacity of human beings; one of the principal ways we reach out to, and orient ourselves relationally in the world as dynamic, self-making creatures who span physical, biological, emotional, cultural, and rational modes of being.

### From Reification to Music-in-(en)action

The enactive perspective has profound implications for how we might begin to (re)conceive of music and music education as a manifestation of life. As I have considered, this is an ontologically continuous and radically non-reductive approach to cognition that embraces complexity, difference, and the feeling-emotional body. It highlights the dynamic, creative, and interpenetrative nature of living meaning-making as it develops through direct embodied experience (Cuffari et al., 2014). As such, enactivism offers a radical shift in perspective from the disembodied, depersonalized, and rather prescriptive conceptions of cognition, knowledge and esthetics we in the West have inherited from Enlightenment thinking (Johnson, 2007). Indeed, it asks us to consider how many of our common epistemological and ontological assumptions may in fact be based in sedimented modes of (dualist) thinking and perceiving that associate ‘meaning’ and cognition solely with language and abstract representation. As I will discuss, such assumptions are problematic when they lead to reductive and reified ways of knowing the world, which often come to prescribe not only how we talk and think about esthetics and science, but also how we think about emotion, nature, or any number of dynamic and transformative phenomena and states of being that we attempt to pin-down with words and categories like love, anger, happiness; or, indeed, education, mind and self (Bai, 2001, 2003).

Reification is seeing the world through conceptual categories which, if not carefully seen through, gives the seer the illusion that reality inherently comes in these categories. Categories are, by nature, discontinuous, dichotomous, linear, and most often, dualistic. Hence in seeing reality through categories, we risk the ability to see the intrinsic connectedness behind all phenomena and phenomenal beings (an ability that ecological consciousness demands). In particular, we risk the ability to see the co-arising of the perceiver and the perceived, the subject and the object (Bai, 2003, p. 8).

Reified notions of music emerge from and reinforce engrained cultural ideologies–such as those surrounding Western classical music’s putative autonomy and superiority, where its universal relevance, meaning and legitimacy is thought to be independent of those who experience and perform it (Small, 1998; Bohlman, 1999; Clarke, 2012). This perspective imposes a reductive, linear, and depersonalized conception of musical communication: there is a musical object that possesses certain objective formal and/or emotional qualities, a performer who interprets and transmits them, and an anonymous subject who perceives them; ‘a view from nowhere and nobody’ (Nagel, 1989; Dibben, 2012). This view places whatever music expresses ‘in the music’ *a priori*; it assumes music to be an objective ‘thing’ rather than an interactive, relational, multi-modal activity; and it creates a rather fixed boundary between some notion of what the music *is* on one hand, and the environments in which it is created and experienced on the other. This conception is especially prevalent in Western academic music education, where the focus remains on producing faithful reproductions of ‘works’ (Elliott and Silverman, 2014); as well as on prescriptive, codified, and hierarchical approaches to analysis and ensemble performance.

Put simply, this perspective has tended to promote an anonymous status for the musical participant; it ignores the necessity of personal agency and embodiment for esthetic experience (Benson, 2001; Bowman, 2004; Dewey, 2005; Johnson, 2007); and it downplays the importance of praxis and personal histories for the development of musical meanings ^7^. As such it is clearly at odds with the enactive approach to cognition when it ignores the interactive, adaptive, and transformative possibilities of the musical organism. And indeed, from this reifying and decontextualized perspective it is not difficult to see why music is often understood to be biologically meaningless–a cultural product or pleasure technology (Pinker, 2009) that plays with our emotional faculties in ways that are not personally relevant (Koelsch, 2013; c.f. Krueger, 2013; Scherer and Coutinho, 2013). Lines (2005) sums up the influence of this perspective on music education well and is worth quoting at length,

The nihilistic state or condition that maintains a ‘valueless’ music (music disconnected from our changing life events) is, however, present in the day to day business of music education. Musical nihilism […] perpetuate[s] a culture of musical impotence, where only a few survive the difficult and detached ride to musical ‘perfection.’ In addition to the affects of such culturally selective traditions, musical nihilism is more generally found in the limited role music plays in the lives of busy urban-dwelling people. To many, the musical sphere is now an area that is forgotten, unused, and neglected. Education in music has come to be seen and regarded by many as an inactive sphere. The conceptual frames–the ways of thinking that support musical inactivity–revolve around several other (not unrelated) key discourses including the cult of the elite musician, music as a commodity-end and the widespread neglect of non-verbal or non-written communication in education. (Lines, 2005)

This orientation has received a good deal of criticism in recent decades, most notably perhaps from music educator, Small (1998), and sociologist, DeNora (2000). DeNora (2000) sees musical meaning as a process that plays out in the evolving ecological, socio-cultural and bio-cognitive contexts of lived experience–music as action, as a therapeutic “force for bio-cognitive organization,” and as part of an esthetic environment through which cultural and individual identities may be constructed and deconstructed (e.g., see Willis, 1978). Small (1998) argues that music is best understood as a verb rather than a noun; his theory of *musicking* considers human musicality as a multi-faceted, relational activity ^8^. This view resonates with the fact that most musical activity around the world is dynamically enmeshed with the activities of life–with work, play, social life, religion, ritual, politics, healing and so on (Blacking, 1976, 1995). In such contexts, music often begins very early in life and is associated with, and often inextricable from, other modes of expressive behavior like dance and storytelling (Green, 2012; Shehan-Campbell and Wiggins, 2013). Here, music retains its status as a transformative communal experience-activity and is meaningful in terms of its relationship to the events and contexts in which it functions (Green, 2012).

Along these lines, a much deeper and more complex conception of what musicality means is emerging in Western scholarship–one that embraces its deep bio-social, ecological and transformative significance for human wellbeing beginning at evolutionary and ontogenetic levels (Cross, 2001, 2010, 2012; Mithen, 2005; van der Schyff, 2013a,b; Croom, 2014). Such research explores how, beginning in infancy, meaningful musical experiences emerge from and support our innate proclivity to seek out and enact meaningful worlds through adaptive embodied kinesthetic interactions with the physical and social-cultural environments (Johnson, 2007; Barbaras, 2010; Gapenne, 2010; Sheets-Johnstone, 2010; Krueger, 2013). For example, musicality is increasingly understood as a form of ‘joint sense-making’ between infant and caregiver, where it is thought to play a major role in the development of empathy and other forms of social cognition (e.g., ‘participatory’ sense-making; De Jaegher and Di Paolo, 2007). For many researchers, this demonstrates the primordial necessity of musicality for embodied and pre-linguistic emotional forms of understanding and communication (Trehub and Nakata, 2001; Parncutt, 2009)–including what Trevarthen (2002) terms the “primary intersubjectivity” that is so necessary for developing social bonds. Such insights resonate closely with the enactive approach to development and cognition; they draw standard dualistic, idealist and objectivist assumptions about the nature and meaning of musical experience in to question; and they place a greater emphasis on understanding how people become involved with music in terms of enacting individual and socio-cultural economies (Green, 2001, 2008)–not as passive listeners, reproducers or ‘consumers’ but rather as autonomous, active and collaborative participants in the construction of meaning (O’Neill and Green, 2004; Reybrouck, 2005; De Jaegher and Di Paolo, 2007).

### Reconciling the ‘Double Articulation’

The enactive perspective also allows us to decentre the Western focus on language, symbol and representation as the fundamental arbiters of communication and meaning. Indeed, because language and music are both auditory modes of communication–and because the great works of Western Classical music appear (post facto) to be constructed largely according to the ‘generative’ or ‘grammatical’ rules of tonal harmony (Lerdahl and Jackendoff, 1996)–a major focus has been placed on the relationship between music and language as cognitive systems (Patel, 2008; Rebuschat et al., 2012) ^9^. This dominant language-centered conception of cognition and meaning has even lead some philosophers to assume that wordless music, as an ‘object’ of perception, cannot properly be understood as meaningful because it contains no semantic content (Kivy, 1990, 2002). Nevertheless, by this view the locus of musical expressivity is still understood to be found in the ‘thing,’ the work itself’ (Small, 1998; Bohlman, 1999; Clarke, 2012); and the esthetic forms of cognition associated with music are often thought to be the product of detached rationalistic (i.e., Kantian) appraisal processes (cf. Johnson, 2007; Scherer and Coutinho, 2013). In brief, such assumptions demonstrate the degree to which seemingly disembodied forms of propositional, conceptual or ‘correspondence-based’ meaning-making associated with language, symbol and representation have become privileged in the modern Western psyche ^10^–thus reinforcing the reified and depersonalized approaches to music I began to critique in the last section.

A useful analysis of this orientation is offered by Eastern thinkers, such as Maruyama (see Nakagawa, 2000), who describe contemporary human existence in terms of a problematic ‘double articulation.’ ^11^ Here the ‘primary articulation’ may be understood in terms of the fundamental autopoietic life processes I discussed above. That is, the dynamic ‘functional circle’ by which an organism enacts a bio-cognitive milieu (*Umwelt*) by means of its receptor and effector systems (von Uexküll, 1973; see also Varela et al., 1993; Reybrouck, 2001, 2005). This biological world is, of course, as real for humans as it is for any other life form. However, humans may also be understood to inhabit a ‘symbolic world’ (the ‘secondary articulation’) that “articulates reality in accordance with its own categories” and that over time comes to be seemingly *“*independent of biological dispositions. In this way, human beings dwell in apparently “double biological and symbolic worlds” (Nakagawa, 2000, p. 38) ^12^. This means that although the biological-somatic articulation is primordial, it nevertheless becomes overshadowed by the dominance of the secondary or linguistic-symbolic articulation–i.e., while “the ‘secondary articulation’ is genetically second” it comes to be “factually and existentially ‘primary”’ in human consciousness (Nakagawa, 2000). Because of this the world often presents itself to us as a collection of seemingly fixed (i.e., reified) independent categories and objective things, pre-given social structures and institutionalized ways of thinking and interacting (Bai, 2013).

According to Johnson (2007), this is exacerbated by the fact that in non-reflective day-to-day life, the body (our ‘biological selves’) tends to “hide out.” That is, how the body tends to retreat to the background as our intentionality is directed ‘out into the world’–while nevertheless tacitly providing the very means and context by which all our perceptions and engagements take place (see also Polyani, 1969; Gallagher, 2005). Such insights have prompted a range of interdisciplinary research that is developing a much more nuanced and embodied view of what communication and meaning-making (musical or otherwise) entails (Searle, 1967; Streek, 1980; Runeson and Frykholm, 1983; Davidson, 2005, 2012; Johnson, 2007; DeNora, 2011). Above all, this work highlights the fact that meaning is not communicated solely through linguistic abstractions, and that action, feeling and lived embodied histories involving participatory forms of sense-making are central to the construction of meaning in creative *living* communication (Sheets-Johnstone, 1999, 2010; De Jaegher and Di Paolo, 2007; Jensen and Cuffari, 2014).

In line with this, one of the goals of the enactive program is to heal the ontological-epistemological gap of the ‘double articulation’ by demonstrating how so-called ‘higher order’ cognitive capacities (e.g., language) may be explained in a continuous fashion through processes such as coupling, autopoiesis, movement and embodied action-as-perception (Stewart, 2010; Froese, 2012). Neurological support for this project comes from the discovery of so-called mirror neurons, which appear to activate both in the brains of those performing actions and in those of onlookers (Gallese and Goldman, 1998; Rizzolatti et al., 2002; see also Tomasello, 1999, 2008). This reinforces the idea of a primordial ‘corporeal intentionality’ where cognition originates in interactive adaptive behavior that involves affectively motivated movement, corporeal articulations, and embodied-empathic states of being (for a brief overview see Thompson, 2007, p. 393–395). Moreover, this research also suggests that while cognition may emerge first through ‘manifest motor activity’ (i.e., actual physical movement and expressions; perceptually guided action), embodied-affective experiences also inform our thinking in ‘covered’ ways ^13^. As Johnson (2007, p. 12) explains, “The core idea is that our experience of meaning is based, first, on sensorimotor experience, our feelings, and our visceral connections to the world; and, second, on various imaginative capacities for using sensorimotor processes to understand abstract concepts” . Thus even in physically inactive and reflective states, our multi-sensory and affectively motivated embodied existence appears to ground how we attribute valences, goals, and intentionality within the social-esthetic environments we inhabit; it underpins our ability to engage in the ongoing process of enacting the meaningful relationships with the people, activities, ideas and things that constitute the complex fabric of our lives as social animals.

### Embodiment, Musical Sense-Making and the ‘Metaphorical’ Mind

Recent studies suggest that mirror-neurons may be spread throughout the brain, implying that they function inter-modally (Leman, 2008; Ramachandran, 2011). This may be considered in the context of further observations, which show that brain areas usually associated with specific bio-cognitive functions may actually engage in cross activation (extreme instances of which result in experiences of synaesthesia; see Ramachandran, 2011) ^14^. This has led a number of researchers (e.g., Lakoff and Johnson, 2003; Johnson, 2007; Ramachandran, 2011) to describe the way we develop understandings of the world in terms of ‘metaphorical’ processes–a notion that goes deeper than the common linguistic-conceptual usage of the term in order to describe the embodied-ecological and often pre-reflective (non-linguistic) processes that allow us to enact meaningful esthetic experiences through the development of cross-modal relations (Eitan and Granot, 2006; Eitan and Timmers, 2010; see also Croom, 2012). Such insights are further supported by a range of research in neuroscience that has demonstrated how cognitive-esthetic potentials depend on the basic bodily systems that allow us to maintain a state of well-being and that constitute the most fundamental ways we become aware of and involved with the world–i.e., metabolism, basic reflexes, the immune system, pain and pleasure responses, basic drives, emotions, and feelings (Di Paolo, 2005; Thompson, 2007; Barbaras, 2010; see also Damasio, 1994, 1999, 2003; LeDoux, 2002). Meaning-making is thus increasingly understood to be based in such complex interactive soma-sensory processes where body and brain, world and mind, form an integrated evolving system. That music deeply affects such processes has been well documented in clinical literature and is, of course, evident in everyday experience (Bunt, 1994; DeNora, 2000; Berger and Turow, 2011; van der Schyff, 2013b; Croom, 2014).

All of this highlights the apparent autonomy of our sensory and metabolic systems while at the same time embracing how they develop co-dependently–that is, how experience and meaning arises relationally (this resonates with the Buddhist conception of the aggregate mind I will discuss shortly). As Johnson (2007) points out, this upsets the rationalizing Enlightenment view that associates esthetics and meaning solely with ‘detached’ forms of ‘higher’ representational or appraisal-based cognition. Rather, from the enactive perspective, our esthetic capacities emerge early in life as the primary way we engage meaningfully with the world. Here, Johnson (2007) draws on Stern’s (1985) notion of ‘vitality affect contours’–a concept that employs embodied-kinetic terms (surging, fleeting, fading away, and so on) to describe how as infants we strive to create a secure, coherent and meaningful existence through primordial cross-modal esthetic processes (see also van der Schyff, 2013b). Such processes, Johnson (2007) suggests, are based on the developmental coupling of the organism and the environment through action; they allow us to recognize and create ‘metaphorical’ relationships between cross-modal perceptions, affective-emotional responses and feelings as we reach out to the world and thus develop and move (and are moved) through time and space. As such, he argues that this primordial embodied-esthetic capacity is the origin of meaning-making and ‘mind’ itself, and therefore grounds all rational thinking and ‘higher’ cognition. Importantly, Johnson (2007) claims that these primordial esthetic ways of meaning-making continue to shape the contours of our experience–and how we meaningfully orient ourselves in the world—even as we grow-up and engage in more abstract, symbolic, categorical or propositional ways of thinking (see also Sheets-Johnstone, 2010; and Nunes, 2010).

In brief, Johnson (2007) argues that traditional ‘cognitivist’ assumptions have led to distorted and reduced understandings of both linguistic and musical communication; and he calls for an approach that allows us to discuss music first in terms of the actual experiences it affords. These, he argues, are grounded in the basic logics of space, time and movement that, via the cross-modal, metaphorical and embodied nature of human cognition, give rise to the fundamental ways we get involved with music within the physical, social, and cultural ecologies we inhabit. Thus, as Johnson (2007) suggests, we would do better to describe musical experience first in embodied-ecological terms such as ‘moving music,’ ‘moving times,’ ‘musical landscape,’ and ‘music as moving force’; or image schemas that describe paths of motion (e.g., source-path-goal; Johnson, 1987; Lakoff and Johnson, 2003; see also Croom, 2012).

From an enactive perspective, then, music is not, first and foremost, an ‘object’ of experience–a ‘thing’ or ‘work’ whose significance is necessarily pre-determined. Nor are musical experiences best understood as fundamentally representational, rule-based or abstract-symbolic phenomena. Rather, they are first sensed as patterns of feeling, emotion and movement; they are experienced directly; and their meanings are enacted in various ways, both shared and personal, by the people, communities and cultures involved (Johnson, 1987; Dewey, 2005). Thus, as Thompson (2007, p. 326) writes, music “has a subjective character that makes it immediately manifest, without observation or inference, as one’s own experience. In this way we experience our listening implicitly, without it becoming an object of awareness.” This insight articulates the need to move beyond the dualistic and objectivist assumptions implicit in many current approaches and embrace a developmental and phenomenological perspective–one that looks beyond mind-world, subject-object dichotomies to explore how the complex, transforming, living, first-person experience of music emerges from its ecologically situated and (inter)subjective nature, and not first from some kind of “object consciousness” (Thompson, 2007, p. 326). Indeed, from this perspective we may better understand how musicking affords the possibility of (re)engaging with the world directly–from the perspective of the somatic-biological ‘primary articulation’ of fundamental life–and thus help to reveal human cognition as an ontologically continuous bio-cultural continuum, whereby meanings developed at the level of symbolic or ‘secondary articulation’ no longer retain a fixed or decontextualized status, but may be seen as continuous with primary forms.

There are numerous musical examples that demonstrate how this is so. However, for the sake of brevity, I ask the reader to consider only one of them–namely, Jimi Hendrix’s ground breaking performance of the ‘Star Spangled Banner’ at Woodstock (see also Clarke, 2005; DeNora, 2011). This performance radically reframes a cultural icon by placing it in the context of a sonic enactment that expresses the brutality of the Vietnam war and the hypocrisy of the hegemonic American foreign policy that supported it. Here Hendrix creates a violent (and ironic) soundscape where the symbol of patriotism incarnate in the Anthem is deconstructed through its interpenetration with chaotic noise–out of which arise sonic episodes that evoke gunfire, falling bombs, and human screaming. What is so remarkable about this performance is not only how Hendrix enacts new understandings of what musical performance and guitar playing entail, but also how his return to the primal cross-modal (‘metaphorical’) origins of music in sound, affectivity, movement, space and empathy allows the evocative ‘musical’ episodes to be experienced as more than simply symbolic. Rather they may be witnessed as transformative phenomena that are lived through (Thompson, 2007)–visceral evocations for critical, emotional, and compassionate imagining that emerge from and simultaneously ‘inform’ and transform the interpenetrative system of performer, listener and the (dissenting) socio-cultural ecology being enacted. Indeed, this wonderful example goes well beyond musical ‘communication as correspondence’ and reveals the meaning of music as a deeply *communal* activity (Krishnamurti, 1970). It also suggests possibilities for new forms of multi-modal (e.g., Kress, 2010) and critically embodied musical analysis (e.g., phenomenological-contemplative) that may have profound pedagogical implications.

### Cosmic Thinking and the Expanding Musical Mind

The enactive position I have outlined above sees musicality as deeply continuous with the primordial embodied-esthetic forms of sense-making that afford a flourishing autopoietic existence. As I have argued, musical experiences entail direct kinds of involvement and understandings that are not always categorical, fixed, or ‘self’ focussed; musical ‘information’ and knowledge is distributed between embodied minds and environments (Sutton, 2006), and emerges via the interpenetration of manifold biological, social, and cultural processes. Thus our musicality affords the possibility of engaging in embodied-ecological forms of knowing that representational modes of consciousness may not be able to afford. And as I began to discuss at the end of the last section, reconnecting with this ‘primary articulation’ as artist-educators may also help us to critically express and examine cultural-political constructions and loosen sedimented ways of thinking beginning at the most primordial levels of sense-making.

For students in an otherwise language and image driven society exploring such possibilities is, of course, extremely valuable. Indeed, because of its deep bio-cultural relevance music has the potential to help us reengage with what Huxley refers to as “first order psychophysical experience” (Huxley, 1965, p. 37). As Bowman (2004) points out, it is precisely music’s ability to make us more aware of the “co-origination of body, mind and culture” that makes is so valuable in education. Unfortunately, the ‘non-verbal humanities’ that are essential for a truly comprehensive liberal education are often not given the attention they deserve in the modern technology driven society (Huxley, 1965). Early childhood music education in the West does tend to encourage such embodied exploration through sound making, improvisation, empathic social enactments, and by maintaining the deep connection between music, dance, song, and storytelling. However, when music student’s move on to formal music education at secondary and post secondary levels they often risk losing this primordial connection to why and how they became involved with music in the first place. The focus shifts to ‘training,’ conformity, authenticity, competition and performing ‘correctly.’ In this way they become ‘conditioned’ by the Western academic model of music making that, as I have discussed, tends to see music as a ‘work’ to be (re)produced or achieved through technical means (Elliott and Silverman, 2014). Far less attention, if any, is given to creativity, embodiment, unique shared and individual experiences, and to understanding what music means for self and society ^15^.

However, as Lines (2005) reminds us, “humans, as music experiencing beings, have the capacity to re-interpret and follow lines of de-territorialisation if they so wish, and in doing so create new musical (and cultural) opportunities of value” (see also Deleuze and Guattari, 1980). This echoes Krishnamurti’s (1970) appeal for humanity to break away from ‘social conditioning’ and the present day pedagogical perspective “aimed at making you conform, fit into and adjust yourself to this acquisitive society […] You are educated to fit into society: but that is not education, it is merely a process which conditions you to conform […].” As *critically* ontological educators, it is our job to understand and share how such conditioning occurs that prescribes the possibilities of being and becoming teachers, musicians and human beings so that we may develop new open-ended pedagogical possibilities. This requires the development of a pedagogical perspective that is non-conformist–one that is much more open and emotionally, empathically, and critically aware; and that takes the unique *communal* experiences of students and teachers seriously as the foundation for fostering an ethical and compassionate world (Jardine, 2012).

With this in mind, Eastern philosophy may have a great deal to offer when it affords practical ways of ‘reawakening’ to richer, animate ontological possibilities (Bai, 2013) than those imposed from the perspective of ‘naive realism’ or what phenomenologists often term the ‘natural attitude’–i.e., the non-critical acceptance of the taken-for-granted ways we come to perceive the world, which lead to sedimented, reified or highly conditioned ways of knowing and being (Husserl, 1960, 1970; Merleau-Ponty, 2002). Like the pedagogical move initiated by Socrates in Plato’s *Republic* (Heidegger, 1998; Thomson, 2001), opening up to the deeper meaning of music through the ‘eastern perspective’ involves a radical ‘turning around’ to face deeper dimensions of reality than the one received by symbolic constructs. As I have discussed, the linguistic-symbolic reality associated with the ‘secondary articulation’ is, of course, an important practical aspect of how we organize our senses in day to day life (Di Paolo et al., 2010; Stewart, 2010). However, as I have also attempted to demonstrate, the belief that this mode of knowing is somehow separate from our embodiment, or is itself wholly constitutive of meaning, is illusory and leads to an impoverished ontology. Without an awareness of more primordial dimensions of existence we may be led to believe that linguistic constructs are something more than signifiers and descriptions of temporary relations between transient categories and things; we may fail to see that they are fundamentally relational and emergent themselves and thus fall into the trap of making false ontological commitments–for example, that “we are living in an objective world of concrete things independent of us” (Nakagawa, 2000, p. 23) ^16^.

Fortunately, music, enactivism and the Eastern perspective can help us open up to a deeper *cosmic reality* that goes beyond semantic articulation and sedimented ways of thinking and knowing. They may reconnect us with the dimensions of the body, feeling and nature–where ‘mind’ emerges as part of fundamental self-organizing life processes (Thompson, 2007; Colombetti, 2014). From this holistic perspective we may begin to understand the “realm of spatio-temporal interconnection in which everything is dynamically and organically interconnected” (Nakagawa, 2000, p. 32). Where nature, life, embodied mind and the universe are,

[…] organic wholes, inseparably connected, and form a cosmic world. The interconnections between things on this level are neither linear causal relations between objective beings nor the fixed codes of meaning of the social world but relationships that are perceived in synchronic mutual causality and interdependence. [T]he cosmic world is not structured in a static manner but in a fluid process of constant metamorphosis. In this sense, it is the world of *Becoming*, which includes both relative being and non-relative being taking place in the flux of self-organizing, self-renewing processes of the universe. Ceaseless processes of birth, growth, decay, and death–the cycle of being and non-being–are the essential aspect of Becoming (the evolutionary process of the universe). (Nakagawa, 2000, p. 32)

Here the grasping for a solid ground in some pre-given reality of fixed things and a stable unified self is exchanged for a ‘groundless’ ^17^ (*sunyata*) universe, where the ever-changing, relational, and interpenetrative experience of being and becoming is embraced as an ‘emergent’ or ‘rising and falling’ phenomenon (Varela et al., 1993). This concept of groundlessness or ‘no-self’ leads to the heart of the Eastern conception of *infinite reality–*the fundamental non-being, non-differentiation, or ‘no-thing-ness,’ from which ‘being’ itself continually emerges and returns to ^18^.

Indeed, a growing awareness of the phenomenon of ‘being’ (thing-ness; identity; thought; the differentiated) as continually emerging from and returning to ‘non-being’ (the born from the unborn; differentiation to non-differentiation; thought into no-thought; life into death) can help us understand that the kind of information, knowledge, understandings and perceptions we develop in real life interactions cannot be properly understood in a strictly functionalist framework because they are not in fact rooted in some kind of substantialist reality ^19^. Rather, as we saw with the enactive approach to bio-cognitive development, they may be better understood as emergent properties of complex relational dynamic systems (Varela et al., 1993)–where ‘entities’ and meanings may be understood as ‘knots’ of various relations, which includes the perspective of the experiencing ‘subject’ herself as constituted by a unique and ongoing history of such relational processes (i.e., the asymmetrical and adaptive co-arising organism-environment relationship I discussed at the beginning). This view decentres the traditional Western preoccupation with categorical fixed (intrinsic or innate) notions of, among other things, identity, drives, emotions, motivations, and intellectual physical and creative abilities (Nakagawa, 2000; Colombetti, 2014). From this perspective such aspects are understood not simply in terms of “something-or-other inside a person” (e.g., pre-given cognitive mechanisms; biological ‘programming’) but rather in the shifting relational context of what happens *between* people and things (Bateson, 1979/1980).

In much of Eastern thinking developing this relational perspective involves a progressive awakening to a multidimensional ontology (see Nakagawa, 2000) that includes:

• the *objective world* (the experience of independently existing things)
• the *social world* (conventions and symbols)
• the *cosmic universe* (the interconnectedness of all things)
• the *infinite universe* (the primordial groundlessness; no-thing-ness)
• the *ultimate reality* (the interpenetration and dependent co-arising of all dimensions)

Here the taken-for-granted conditioning associated with the world of fixed objects and socio-cultural symbols and constructs comes to be understood as such through a growing awareness of the deep interconnectedness, co-dependence and impermanence of all things and experiences.

To be clear, this is not to say that as one begins to gain an awareness of the cosmic and infinite dimensions of reality the objective and cultural worlds are negated. Rather, like the philosopher returning to the cave, one learns to reengage with them with a new understanding of what they really are. Thus an awareness of *ultimate reality* involves awakening to the fact that the experience of all ‘independent’ things–culture, nature, music, social relations, emotions, feelings, identity and self, mind and world–are relational and transforming; they ‘rise and fall’ through interpenetrative relations. Experience of the world is thus an enactive or dependently co-arising phenomenon ^20^ (Macy, 1991; Varela et al., 1993; Nakagawa, 2000).

In brief, the Eastern perspective offers a framework whereby one may begin to engage in new ways of experiencing and understanding from a deeper, interpenetrative and transforming multidimensional ontological perspective. In an educational context this opens up critical and hermeneutic possibilities, where the interactive and autopoietic nature of being and becoming comes to the fore (Kincheloe, 2003; Jardine, 2012; Seidel and Jardine, 2014), and where students and teachers may move beyond externally dictated and sedimented ways of understanding and engage more authentically, reflectively, and compassionately with the world and themselves. Here presuppositions may begin to be questioned, conditioning may be better understood, and teachers and students may get down to the business of exploring musical development in a way that embraces the organic processes of life and the primary embodied-ecological articulation of human consciousness–which reflects their fundamental status as living, autonomous participatory sense-makers (De Jaegher and Di Paolo, 2007). From this perspective music education becomes a communal (Krishnamurti, 1970) process that reflects the multi-modal and bio-cultural nature of music-as-action–where subject–object dualities and mechanistic metaphors recede; where transformative, embodied, non-verbal and affective-esthetic ways of knowing come to the fore; and where the unique and transforming relationships between students, teachers and the musical phenomena being explored may be experienced in context of a dynamic, living pedagogical ecology.

### Exploring the Buddhist Psychology of ‘Self’ for Music Education

The enactive approach to cognition resonates deeply with the Buddhist mindful-awareness tradition when it claims that although phenomenal distinctions can be made between the objects of experience, nothing, including the ‘self’ or the ‘mind,’ exists as an independent entity–all experience, things, thoughts and ideas arise and evolve co-dependently (Murti, 1955; Hopkins, 1983; Gyamtso, 1986; Kalupahana, 1987). Interestingly, the juxtaposition of mindful-awareness with the enactive approach to cognition also echoes important contemporary scientific concerns regarding the notion of ‘self’–where the mind is understood as an essentially pluralistic phenomenon (Varela et al., 1993). The work of Minsky (1986) and Jackendoff (1987) in cognitive science are particularly notable here as they are both willing to follow this insight to the threshold of experience to discover, as Hume (1964) and others have before them, that the notion of a fixed and unified cognizing subject appears to be a phantom (see Varela et al., 1993). Jackendoff (1987) draws out the multi-modal nature of cognition and the non-unified nature of the cognizing subject. And likewise, Minsky (1986, 39–40) suggests that the self should not be understood as a “centralized and all-powerful entity, but as a society of ideas that include both the images of what the mind is and our ideals of what it ought to be.”

Indeed, this comes close to “the Buddhist distinction between the coherent pattern of dependently originated habits that we recognize as a person and the ego-self that a person may believe she has and constantly grasps after but which does not actually exist” (Varela et al., 1993, p. 124). However, while the mindful-awareness tradition makes the distinction between the representation or concept of a fixed ego-self and the habitual ways of thinking that lead us to grasp for it (Gyamtso, 1986), no such distinction is clearly articulated in cognitive science. That is, traditional cognitive science discovers the disunity of the mind-self in the hypothezised sub-personal mechanisms (e.g., adapted modules) associated with the cognitivist information-processing approach; but because it possesses no disciplined method for examining experience it can go no further than this ^21^. By contrast, the mindful-awareness tradition begins not with hypotheses, but with a rigorous examination of lived experience. It thus offers a systematic way of developing consciousness that begins with sensation, movement, feeling, as well as the needs, desires and cravings that motivate cognition as action.

From this perspective experience is attended to in the context of an interpenetrative aggregate consciousness (or ‘self,’ see below) that may be further specified according to how experience is sourced in the senses (which are themselves understood as ‘minds’; see Varela et al., 1993). Thus the mindful-awareness practitioner is interested in examining the movements of the ‘aggregate mind’ from the first person perspective–not in order to grasp some fixed notion of it, but rather to become increasingly aware of consciousness as an ongoing emergent and transforming process. This involves moving awareness beyond its habitual state toward the possibilities that emerge when more fundamental, embodied-affective, ‘metaphorical,’ and interpenetrative aspects of consciousness and cognition are recognized and developed (a possibility not entertained by the traditional cognitivist who relegates all basic cognitive processes to the sub-personal level). As I discussed above, this allows us to embrace plurality and difference in terms of a continually transforming multi-dimensional, organic, *cosmic* whole.

With this in mind, Buddhist psychological concepts associated with the aggregate mind (no fixed or unified self) may hold great potential in for music education–especially in instrumental instruction and ensemble practice. As Nakagawa (2000) points out, these concepts are not meant to be taken as religious dogma (see also Garfield, 1995), rather they are simply offered as psychological (and therapeutic) frameworks, which may be developed in various contexts in order to better understand embodied experience; the manifold aspects and transforming nature of the ‘self’; our relationships with people and things in the world; and how certain ways of thinking and doing may lead toward suffering in ourselves an others. In this way, they provide highly pragmatic ways of understanding the psychology of personal musical engagement in a multi-dimensional context from moment to moment, day to day, and over the course of a lifetime.

For example, when I am working with students I notice that they often struggle with some notion of what they think the music should be and how it should be realized. Sometimes this notion can be very vague, which results in feelings of inadequacy and confusion (a grasping for stable ground in some external authority). Other students arrive with a highly conditioned point of view that resists and restricts other possibilities–which may lead to arrogance or closed mindedness (clinging to sedimented conceptions of self, music and the world). Either way, there is nothing enjoyable, revealing or transformative about such closed and prescriptive orientations. Indeed, by focussing on conforming to some fixed outcome, musical identities and meanings become defined by external norms. This results in reducing student–teacher (and student–student) communication to mechanical technical issues and plays down transformative communal possibilities–the enactive, self-world making potentials of musicking are pushed aside in order to render the music (and musician) as a product that must conform to some kind of externally driven standard ^22^.

In order to help understand and move beyond such conditioning, I often ask students to attend to and discuss musical experiences according to the interacting categories associated with the “five aggregates” (see Varela et al., 1993). With practice students can learn to attend closely to these dynamic aspects of self and become more aware of how the relationship between them changes and develops.

(1) Forms (*rupa*) – the physicality (primordial embodiment) of the body-instrument-world relationship.
(2) Feeling and Emotion (*vedana*) – the transforming affective contours of musical action and meaning.
(3) Perception, impulses and cognition (*sanna*) – the primary discernment and ‘directedness’ of musical relationships (intentionality).
(4) The will and volition (*sankhara*) – the dispositional formations (habits) and desires that motivate musicking.
(5) Consciousness of all aggregates (*vinnana*) – musical discrimination as continuous across the aggregates; the sense of a binding relationship.

This may be developed further through an exploration of the Buddhist framework through which all co-dependent arising occurs (in a musical context see Lowe, 2011). This is often simplified to include twelve main states of being or *nidanas* (see Nakagawa, 2000).

(1) ignorance (*avidya*)
(2) volitional (karmic) formation (*samskara*; the fourth aggregate)
(3) consciousness (*vijnana*; the fifth aggregate)
(4) name and form (*nama-rupa*)
(5) the six senses (*sadayatana*)
(6) impression-contact (*sparsa*)
(7) feeling (*vedana*; the second aggregate)
(8) craving (*trsna*)
(9) grasping (*upadana*)
(10) becoming (*bhava*)
(11) birth (*jati*)
(12) aging, dying, grief, suffering (*jaramarana*)

These descriptive-reflective frameworks resonate in many ways with the autopoietic and pluralistic/co-arising perspective on “mind in life” (Thompson, 2007) I discussed at the outset. However, they need not be seen as necessarily referring to a ‘life-span’ in the literal sense. Indeed, they may also be understood in a ‘circular’ fashion in order to examine the emergent or ‘rising and falling’ nature of contextual experience from moment to moment; to explore the conditioning that leads to certain desires, craving and grasping; and thus develop conscious awareness beyond its taken-for-granted state.

When practicing a difficult passage of music, a challenging instrumental technique, or a new concept, these frameworks may help students better understand and discuss the learning process from the perspective of direct embodied experience; to explore how their consciousness–their ‘self’–is really a transforming multi-dimensional phenomenon. Learning new musical activities exercises the aspects of the self, which are required to reach out to and engage with the world and each other in new ways. Initially the results are never harmonious and may involve a kind of discomfort (Sudnow, 1978). In such situations the aggregates seem somewhat alienated from each other and a frustrated desire and grasping may dominate–an uncomfortable perceptual closing in on the self and away from the world where the emotional and motivational aspects of the self may find themselves in crisis. Here a caring (encouraging, compassionate; Noddings, 2012) teacher who has herself worked through similar experiences may be of great help. She may encourage the student to open up to their experience and attend to this process carefully and honestly. This may help the student become more aware of how musical knowledge develops in the context of her unique bio-cognitive economy; and how certain sedimented assumptions–bodily and cultural conditioning–may be preventing her from engaging fully with the possibilities afforded by the situation. The student may then begin to recognize how new levels of musical being may be developed as the aggregates are reintroduced to each other in a new ways–and thus begin to let go of the taken-for-granted notions of self, music and meaning and open up to a relational way of musicking that values imagination, creativity and personal transformation.

Such a process reinforces the dynamic and interpenetrative (autonomous, dynamically open; see above) relationship between student and teacher. The teacher is attentive to and cares for the unique needs of the student; she shares her knowledge through example and supports the student’s reaching out to becoming-musical by fostering an environment that affords the discovery of new dynamic relationships and patterns (Granott and Parziale, 2002; Noddings, 2012). As they go, student and teacher may then explore things together from more nuanced emotional, bodily and conceptual points of view. What is discovered here is that there may be many ways to approach and understand the given piece, passage, technique, concept, or musical cultural meaning–a perspective that suffices or is viable in one context (or on one day) may not work well in another. Students thus learn to let go of fixed understandings and attend to the moment; they are encouraged to reach out for difference, transformation and they learn to avoid relying on facile or simplistic recognitions of musical relationships that emerge from conditioned, reductive or reified understandings (Dewey, 2005).

Along these lines, Biswas (2012) discusses how improvisation and the bodily exploration of repetitive musical activities (e.g., rhythmic drumming) may be combined with mindful awareness techniques (in his case *vipassana* meditation) in order expand musical awareness. Here practitioners are asked to attend to their entire bodies (breathing, extremities, parts of the body not explicitly involved in the action) as they engage in musical activities, and thus better understand the bodily conditioning (tensions, grasping, ways of doing and feeling) that may be preventing them from developing new understandings and potentials ^23^. Such activities may be combined with other forms of exploration such as Alexander technique, phenomenological and multi-modal analysis (e.g., metaphorical description); and they may also be explored collectively in ensembles where students may be encouraged to extend their listening and action out into the world–to reach out empathically and compassionately and thus participate more deeply in the communal-interpenetrative mind and world-making process that music affords. Here too, conditioning and dependent co-arising may explored at the macro level and thus musicking, as Biswas (2012) suggests, may be understood as a form of “public meditation” (see also Sarath, 2006).

Developing these basic psychological frameworks through musical practice may also extend well beyond the practice room and into the life-world of the student. That is they may help students better understand what it means to be a dynamic, creative, autopoietic creature–as well as the suffering and psycho-spirtual or creative ‘death’ (i.e., dynamic closure) associated with grasping for unchecked desires and predetermined outcomes (fame, money, entitlement), and fixed or detached conceptions of self and world. Developing such awareness may help them avoid the pitfalls of pride, competitiveness and bitterness as they go on to experience both the successes and the inevitable personal frustrations and disappointments they will face throughout their career. This may also help them better understand how such grasping may affect their performance and enjoyment of music in the moment as they learn to let go and attend more deeply to what they are doing and feeling–to keep the music and themselves dynamically open, compassionate and thus, creatively ‘alive.’

## Conclusion

From the Eastern-enactive perspective, music is not retrieved from a pre-given world ‘out there’ but rather emerges from our embodied consciousness as it reaches out to, transforms, and is transformed by the ongoing process of empathic inter(en)action with objects, ideas and other minds. By this view, the challenge of becoming a skilled (and compassionate) musician and music educator is not due to the fact that the music is ‘hard’ but rather because it is ‘open,’ equivocal and transformative (Bowman, 2004). And indeed, we might also note here how the act of listening arguably offers a more explicitly transformative and interpenetrative perspective on the world than the visual dimension usually affords. Through vision the differentiation (separation) or fixity of things, objects or events is more easily taken for granted. The auditory world, by contrast, is less obviously differentiated and more explicitly interpenetrative, affective and emergent–where events continuously rise and fall from a background of ‘silence’ or non-differentiation in ways that are co-dependent with our unique ways of reaching out through listening (Ihde, 2007). With this in mind, students and teachers may be encouraged take their active listening ‘out’ into the urban and natural worlds–creatively and reflectively–in order to better understand the origins of music as an active ecological phenomenon and thus develop a deeper awareness of the primordial, empathic, and interpenetrative experience of being-in-the-world. Mathews (2008, p. 53) develops this pedagogical possibility when she points out how empathic and ‘synergistic’ activities such as musicking may open us up to a deeper compassionate relationship with all life and thus help “induce in humans a moral point of view with respect to other-than-human life forms” (see also Nollman, 1990, 1999, 2000; Wallin et al., 2000; Rothenberg, 2005, 2014; Rothenberg and Ulvaeus, 2009). Here students and teachers may be “encouraged to identify imaginatively with wider and wider circles of the [sonic] landscape, until, hopefully, the students acquire an expanded sense of identity, described in deep ecology literature as the ‘ecological self”’ (Mathews, 2008, p. 54; see Naess, 1985, 1995). In this way our musicality may reawaken us to the deep continuity between natural and human worlds–a continuity that, as I have argued all along, is beautifully spanned by the bio-cultural nature of music itself.

There is, of course, much more to say about the relevance of enactivism and the Eastern perspective for being and becoming a musician and music educator. However, I hope I have offered the reader a useful introduction and provided some possibilities for how music and music education may be better understood a ‘manifestation of life.’ As I have attempted to show, this perspective sees music education as a deeply contemplative activity that begins with an exploration of the primordial meaning of musicality as an open intersubjective form of empathic participatory sense-making–one that originates deep in our biological selves. I hope, then, that the ideas and concepts I have offered here may help music educators become more aware of the autopoietic and world-making potentials of music; move beyond nature–culture dichotomies as well as nihilistic and purely technically driven approaches; and thus reengage with music and music education as a powerful way of reconnecting human life with the fundamental life.

## Footnotes

1 Other thinkers have coined similar phases with the goal of developing a ‘global’ holistic philosophy of life that incorporates the best of Eastern and Western perspectives, as well as indigenous knowledge from around the world (see Nakagawa, 2000).

2 For a review of enactive music cognition see Matyja and Schiavio, 2013.

3 This view has an antecedent in Dewey’s (1938/1991, p. 26) principle of continuity where “rational operations grow out of organic activities without being identical with that from which they emerge.”

4 Critical pedagogue Kincheloe (2003) echoes this insight when he writes, “In both its corporeal and cognitive expressions the autopoietic life process reaches out for difference, for novelty, to embrace its next ontological level.”

5 However, once such asymmetry (i.e., the permeable boundary and metabolic processes that occur inside it) can no longer be supported the organism-environment relationship, like any other complex dynamic system, dissolves into non-differentiation. This may come about through a disruption in the ability of the organism-environment coupling to engage in the dynamic, adaptive behavior necessary to maintain a viable life-world, which finally leads to sickness and death. As Di Paolo and Thompson (2014, p. 73) write, “Basic cognition, on this view, is not a matter of representing states of affairs but rather of establishing relevance through the need to maintain an identity that is constantly facing the possibility of disintegration. From this perspective, the body is not just the means but also an end of being a cognitive system. To put the point another way, basic cognition is more a matter of adaptive self-regulation in precarious conditions than abstract problem solving.”

6 As Johnson writes, “meaning includes patterns of embodied experience and pre-conceptual structures of our sensibility (i.e., our mode of perception, or orienting ourselves and of interacting with other objects, events, or persons). These embodied patterns do not remain private or peculiar to the person who experiences them. Our community helps us interpret and codify many of our felt patterns. They become shared cultural modes of experience and help to determine the nature of our meaningful, coherent understanding of our ‘world”’ (Johnson, 1987, p. 14).

7 This point of view also shows itself in the broader consumer culture where musical conventions, the score (in both the physical and the abstract sense) and, more recently, the recorded product, become what is signified by the word ‘music.’ Along these lines, a number of critical theorists have pointed out how this attitude is in line with the continuing process of reification associated with the development of mechanical reproduction and commodity fetishism in Western capitalist society (Lukács, 1971; Adorno, 1973; Adorno and Horkheimer, 2002; Benjamin, 2008).

8 Small (1998, p. 9) writes, “The fundamental nature and meaning of music lies not in objects, not in musical works at all, but in action, in what people say and do. […] To music is to take part, in any capacity in a musical performance, whether by performing, by listening, by rehearsing or practicing, by providing material for the performance (what is called composing), or by dancing.”

9 Along these lines, some psychologists argue that human musicality is biologically meaningless; that it is dependent, or ‘parasitic’ on, cognitive information processing mechanisms that evolved to support language (e.g., syntax, auditory scene analysis and so on; Sperber, 1996; Pinker, 2009; c.f. van der Schyff, 2013a).

10 As Johnson (2007, p. 216) writes, “On one side of the dualistic gap we have concepts, thought, reason and knowledge. On the other side we have sensations, feelings, emotions, and imagination. What has been so fateful about this dualism for contemporary philosophy is the way it *aligns meaning with the cognitive and thus dismisses quality, feeling, and emotion from any account of meaning.”*

11 A similar insight is shared by Cassirer (1944): “Man has, as it were, discovered a new method of adapting himself to his environment. Between the receptor system and the effector system… we find in man a third link which we may describe as the *symbolic* system. This new acquisition transforms the whole of human life.

12 Enactivists make similar distinctions between ‘first’ and ‘second’ order sense-making (Froese, 2012).

13 For example, Molnar-Szakacs and Overy (2006; Molnar-Szakacs et al., 2011) have developed an action based perspective on musical communication they refer to as the shared affective motion experience model (SAME). Put very simply, this model offers a way of understanding how the proposed mirror neuron system in humans allows music to access emotional faculties: depending on the listener, movement is ‘extracted’ at different levels of the motor system; and emotional intentions are thought to be inferred directly from this motor-activity, whether actual or simulated.

14 This process does not involve the neat sharing of resources via the inputs and outputs of ’cognitivist’ computational modules, but rather a more plastic process whereby neural ‘webs’ develop in complex interpenetrating ways that allow for deep and sometimes highly ideographic relationships to form between seemingly disparate areas of experience (e.g., color and number, sound and texture; see Ramachandran (2011).

15 Lines (2005) writes, “The problem with technically driven conceptions of music is that they fail to adequately attend to the changing and transient character of music events. Rather, the technicism of teaching methods that reinforce reductive conceptions of music obscures music’s relationship to humankind and the qualities of musical experiences that engage the character of that relationship.”

16 This resonates with James’ famous dictum: “[…] nothing real is absolutely simple, that every smallest bit of experience is a *multum in parvo* plurally related, that each relation is one aspect, character, or function, way of its being taken, or way of its taking something else […]” (James, 1996).

17 It is interesting to note here that the ancient recognition of the inability to find stable, objective, or pre-given epistemological foundations, either ‘internally’ or ‘externally,’ associated with the Buddhist conception of *sunyata* actually prefigures much so-called continental philosophy–where ‘groundlessness’ is often assumed to be a distinctly post-modern issue associated with scientific and cultural developments in the 20th century (Rorty, 1979; Putnam, 1987; see Varela et al., 1993).

18 A number Eastern thinkers have discussed how the void tends toward self-organization–i.e., how the universe itself possesses a primordial autopoietic tendency (Nakagawa, 2000). Interestingly, such processes have been explored with computer modeling where virtual structures self-organize from milieu whose initial state is random and non-differentiated (see Varela et al., 1993; Thompson, 2007).

19 This is echoed from the enactive perspective by Froese (2012): “[…] the living body is the ultimate source of significance, but it can only serve this role because it is a continually actively constructed body that is always precarious and vulnerable to disintegration. The core meaning that derives from the ever-present potential of death, an eventuality which can be negatively defined as the cessation of all functioning, cannot adequately be captured in functionalist terms” (see Di Paolo et al., 2010).

20 Dependent co-arising is often understood in a complex three dimensional framework that begins to be explored in the thought of Nagarjuna and that is further developed in the ontology of Hua-Yen Buddhism (see Suzuki, 1970; Chang, 1971; Garfield, 1995; Tachikawa, 1997; Nakagawa, 2000).

21 As Varela et al. (1993, p. 54) write, “Jackendoff assumes that everyday–largely mindless–experience provides access to all relevant phenomenological evidence and that the phenomenological quest is limited to just that largely mindless state. He considers neither the possibility that conscious awareness can be progressively developed beyond its everyday form (a strange omission given his interest in musical cognition) nor that such development can be used to provide direct insight into the structure and constitution of experience.”

22 This is not to imply that musical traditions and conventions are to be ignored or rejected. To the contrary such an approach seeks to vitalize traditions by encouraging students and teachers to engage critically and creatively with them as autonomous world-makers–rather than passively allowing them to externally impose fixed ways and meanings.

23 As a drum teacher, I often work with students in this way with repeating polyrhythms, where students are asked to attend to and describe the transforming experience of listening and playing from a range of embodied-esthetic perspectives.
